# Risk factors and preventive strategies for post-traumatic stress disorder in neonatal intensive care unit

**DOI:** 10.3389/fpsyg.2022.1003566

**Published:** 2022-11-09

**Authors:** Maria Di Chiara, Gianluigi Laccetta, Sara Gangi, Benedetta De Santis, Caterina Spiriti, Martina Attenni, Laura Bertolaso, Giovanni Boscarino, Maria Chiara De Nardo, Gianluca Ciambra, Pasquale Parisi, Gianluca Terrin

**Affiliations:** ^1^Department of Maternal and Child Health, Policlinico Umberto I, Sapienza University of Rome, Rome, Italy; ^2^Department of Neuroscience, Mental Health and Sense Organs (NESMOS), Faculty of Medicine and Psychology, Sant’Andrea University Hospital, Sapienza University of Rome, Rome, Italy

**Keywords:** PTSD, PSS:NICU, preterm, parents, newborns, Kangaroo-Care, gestational age, anxiety

## Abstract

**Background:**

Preterm birth and admission to the neonatal intensive care unit (NICU) could induce post-traumatic stress disorder (PTSD). PTSD is an important factor to focus on, as it is associated with parental mental health difficulties and with changes in caregiving quality such as increased intrusiveness, reduced sensitivity, and increased attachment insecurity for the child.

**Aims:**

We aimed to study the main risk factors, in the early life of newborns, and preventive measures for PTSD in parents of neonates hospitalized in the NICU.

**Methods:**

We included parents of preterm newborns, consecutively admitted to the NICU of the University La Sapienza of Rome. The presence of PTSD following preterm birth and NICU admission was assessed using the Clinician-administered PTSD scale (CAPS) at enrollment and at 28–30 days following NICU admission or the moment of discharge. We also evaluated the Family Environment Scale which measures the social environment of all types of families; the Parental Stressor Scale which measures parental anxiety and stress; the Spielberger State-Trait Anxiety Inventory consisting of two parts measuring the State (response to present situation) and Trait (pre-disposition to be anxious) anxieties separately, and the Beck Depression Inventory Second Edition assessing depressive symptoms.

**Results:**

We found, in a multivariate analysis, that the gestational age of newborns admitted to NICU significantly (β = 2.678; *p* = 0.040) influences the occurrence of PTSD. We found that the cases showed significantly (β = 2.443; *p* = 0.020) more pathological Parental Stressor Scale sights and sounds scores compared to controls. The early Kangaroo-Care (KC) significantly (β = −2.619; *p* = 0.015) reduces the occurrence of PTSD.

**Conclusion:**

Post-traumatic stress disorder in parents of preterm newborns is a pathological condition that should be properly managed, in the very first days after birth. The NICU environment represents a main risk factor for PTSD, whereas KC has been demonstrated to have a protective role in the occurrence of PTSD.

## Introduction

Post-traumatic stress disorder (PTSD) is a pathological condition included in the Diagnostic and Statistical Manual of Mental Disorders (DSM-5) (Vahia, 2013). Exposure to traumatic events, including the threat of death or serious injury to the individual or another, that is accompanied by feelings of horror, helplessness, or intense fear, induces PTSD.

The clear association between PTSD and NICU admission is still unclear because of the lack of a standardized assessment of parental stress. Preterm birth and admission to the neonatal intensive care unit (NICU) could be highly traumatic events for the parents (Holditch-Davis et al., 2003). Lower gestational age (GA) is associated with an increased incidence of complications and mortality. The extreme prematurity, therefore, may worsen parents’ perception due to the unexpected conclusion of their pregnancy because they may have not been able to prepare themselves for separation from the baby (Zanardo et al., 1998; Brisch et al., 2003).

Parenting stress is an important factor to focus on, as it is associated with parental mental health difficulties and with changes in caregiving quality such as increased intrusiveness, reduced sensitivity, and increased attachment insecurity for the child (Holditch-Davis et al., 2003).

Neonatal intensive care unit is a highly technological and medicalized clinical facility developed to grant the survival of the most fragile newborns. Alongside the survival of preterm newborns, parents’ issues, related to hospitalized newborns, arose and drove attention to the nature of parents’ stress. NICU is a critical environment to provide the best care for preterm neonates. Environmental stressors include the appearance of their critically ill infant with a variety of tubes and intravenous lines, surrounded by technological equipment, such as respirators and monitoring equipment. Moreover, additional sources of stress include the persistent overall sights and sounds of the unit (Miles et al., 1993; Miles and Holditch-Davis, 1997). It has been described that the NICU environment disrupts the parents’ involvement in infant care and jeopardizes the attachment process between parents and babies (Borghini et al., 2014).

We previously demonstrated that alteration of parental role increased the risk of PTSD and that familiarization with the NICU environment and increasing participation of the parents in the care of neonates, including the kangaroo care (KC), during the first weeks of life improved parental role perception (Gangi et al., 2013). KC is an intervention that can improve the involvement of the parents in the care of the babies.

During KC, the infant is placed in skin-to-skin contact with the mother’s, father’s, or caregiver’s chest in a frontal position with the infant’s head turned sideways (Nyqvist et al., 2010). KC is known to be among the most effective interventions for preventing death in infants with low birth weight (WHO Immediate KMC Study Group, 2021).

Many research studies have identified the positive effects of skin-to-skin contact such as sensory stimulation on the growth and behavioral development of preterm infants. It has been demonstrated that KC influences the risk of maternal postpartum depressive symptoms; however, the effects of this intervention on PTSD of both parents are largely unexplored. It is well described that NICU admission represents a traumatic event, however, the explanations of how it belongs to parents’ PTSD are inconclusive.

Starting from these considerations, we aimed to study the main risk factors and protective measures, such as KC, for PTSD in parents of neonates hospitalized in the NICU.

## Methods

We consecutively included parents of newborns, with gestational age (GA) ≤ 37 weeks admitted to the NICU of the University La Sapienza of Rome over 3 months.

The presence of PTSD diagnosis following preterm birth and NICU admission was assessed using the Clinician-administered PTSD scale (CAPS) at 30 days, based on the consensus opinion of two patients trained in PTSD (Weathers et al., 2018). Besides, we also administered questionnaires at the enrollment, within 5 days from the date of birth, to collect symptoms associated with a post-traumatic event and assess factors related to the acute event.

We excluded parents of newborns with life-threatening conditions in the first 24 h, congenital abnormalities, parents less than 18 years of age, drug-addicted, with insufficient Italian language proficiency, with a known psychiatric diagnosis, or with a previous preterm birth/NICU-admitted infant and parents whose infants were transferred to another care facility before 30 days.

Two members of the research staff, a trained neonatologist and a trained psychologist, administered the questionnaires to all the enrolled parents (mothers or fathers); data collection and administration of the questionnaires took place in a designated room inside the neonatal ward of Sapienza University of Rome. Clinical data collected by researchers were different from clinicians. We discussed and defined a protocol for the collection, measurement, and interpretation of data before starting the study. A third-party observer was involved to collect data. Finally, a blinded statistician performed the data analysis. The KC intervention was conducted according to our local protocol (Supplementary material). The Family Environmental Scale (FES) (Weathers et al., 2018) the Parental Stressor Scale: Neonatal Intensive Care Unit (PSS:NICU) (Miles et al., 1993), the Spielberg State-Trait Anxiety Inventory (STAI), and the Beck Depression Inventory–Second Edition scale (BDI-II) were administered at the enrollment (Strunk and Lane, 2016; Weathers et al., 2018; Zsido et al., 2020). In the Supplementary material, we reported the description of each scale measure (Supplementary material). All enrolled parents were divided into two groups, by two trained psychologists who administered PTSD scales, unaware of the study aims: the first group included parents that developed PTSD; and the second group included parents without PTSD diagnosis according to the previous standardized criteria.

## Ethics

The study was conducted in accordance with the World Medical Association Declaration of Helsinki for medical research involving human subjects. All the subjects provided written informed consent before enrollment. We collected anonymized data after written informed consent was obtained from the parents of each enrolled infant. Data are available on request from the Department of Maternal and Child Health Policlinico Umberto I Hospital, La Sapienza University of Rome, Italy.

## Results

A blinded statistician performed data analysis using Statistical Package for Social Science Software (SPSS Inc., Chicago, IL, USA), version 25.0.

We enrolled 82 parents, 22 with the diagnosis of PTSD as cases and 60 parents without the diagnosis of PTSD as controls. We checked for normality using the Shapiro–Wilk test. We used the χ^2^ test for categorical variables and the *t*-test, Mann–Whitney, or McNamara for paired and unpaired variables.

The main demographic and clinical characteristics, at baseline, of participating cases and controls were summarized in Table 1. Baseline clinical characteristics were similar between cases and controls. We did not find any difference between the rate of fathers and mothers. We found that the frequency of foreign parents was significantly (*p* = 0.020) higher in controls compared to cases (Table 1). The rate of history of both depression and anxiety was significantly (*p* < 0.01; *p* = 0.016) higher in cases than in controls (Table 1). Parents with the diagnosis of PTSD showed a significantly higher rate of newborns with GA ≤ 32 weeks, compared with controls. Parents with PTSD showed a significantly (*p* < 0.001) reduced percentage of early KC compared to controls (Table 1).

**TABLE 1:** Clinical characteristics of the study population.

	Cases	Controls	*P* value
		
	*n* = 22	*n* = 60	
***Parental characteristics***			
Parents’ age°	16 (72.7)	26 (43.3)	*0.017*
Parental bachelor’s degree	9 (40.9)	15 (25.0)	*0.0130*
Previous child	3 (13.6)	13 (21.7)	*0.318*
Mother	17 (77.3)	36 (60.0)	*0.116*
Native Italian	19 (86.4)	36 (60.0)	0.020*
Single mother	0 (0.0)	1 (1.7)	*0.732*
Married status	15 (68.2)	47 (78.3)	*0.251*
Family psychiatric disease	3 (13.6)	7 (11.7)	*0.536*
History of depression	7 (31.8)	1 (1.7)	<0.001*
History of anxiety	10 (45.5)	11 (18.3)	0.016*
***Obstetrics characteristics***			
Primigravida	9 (40.9)	33 (55.0)	*0.189*
Assisted conception techniques	4 (18.2)	18 (81.8)	*0.114*
Smoke in pregnancy	4 (18.2)	10 (16.7)	*0.553*
Emergency delivery	21 (95.5)	54 (90.0)	*0.391*
Diabetes	2 (9.1)	3 (5.0)	*0.407*
Hypertension	6 (27.3)	11 (18.3)	*0.276*
IUGR^a^	3 (13.6)	9 (15.0)	*0.593*
PROM^b^ > 18 h	1 (4.5)	11 (18.3)	*0.108*
Twin pregnancy	5 (22.7)	17 (28.3)	*0.419*
Cesarean section	16 (72.7)	50 (83.3)	*0.220*
***Postnatal data***			
GA^c^ ≤ 32 *weeks*	20 (90.9)	28 (46.7)	<0.001*
Male sex	11 (50.0)	18 (30.0)	*0.079*
Apgar score at 5’	8.05 ± 0.899	8.03 ± 1.248	*0.967*
BW^d^, *grams*	1336.09 ± 406.849	1466.33 ± 438.163	*0.228*
Early Kangaroo-Care	2 (9.1)	40 (66.7)	<0.001*
Parenteral nutrition	19 (86.4)	49 (81.7)	0.447

°Parents’ age ≥ 35 years old. ^a^IUGR, intrauterine growth restriction. ^b^PROM, prolonged rupture of membrane. ^c^GA, gestational age. ^d^BW, birth weight. Data were expressed as mean ± standard deviation, when not specified. *p < 0.001. Italic values represent the significance of *p*-value.

**FIGURE 1:**
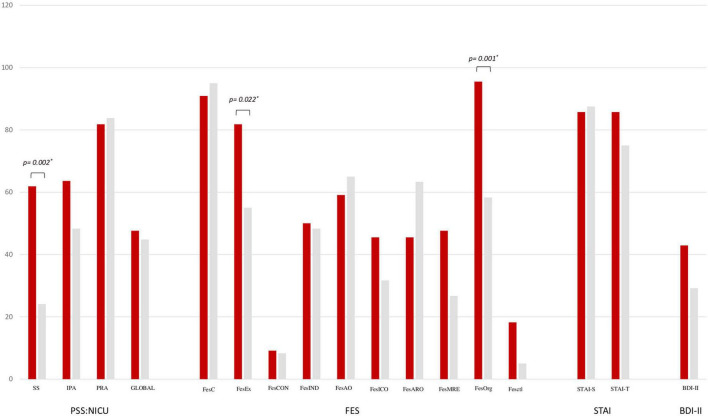
Parental stressor scale: Neonatal intensive care unit (PSS:NICU), family environment scale (FES), spielberg state-trait anxiety inventory (STAI), and beck depression inventory–second edition scales (BDI-II) between cases and controls. PSS:NICU scale: sights and sounds (SS), infant behavior and appearance (IBA), parental role alteration (PRA), PSS global. FES scale: (1) Relationship dimensions: cohesion (FesC), expressiveness (FesEx), and conflict (FesCON); (2) Personal growth dimensions: independence (FesInd), achievement orientation (FesAO), intellectual-cultural orientation (FeslCO), active-recreational orientation (FesARO), and moral-religious emphasis (FesMRE); (3) System maintenance dimensions: organization (FesOrg) and control (FesCtl). STAI: state anxiety (STAI-S) and trait anxiety (STAI-T). *p < 0.01.

In Table 2, the main morbidities related to prematurity, within hospital stay, of enrolled parents’ newborns are reported (Table 2). No statistically significant difference was found between cases and controls (Table 2). In Figure 1, we reported the results of scale items. We found that the cases showed significantly more pathological PSS:NICU sights and sounds (61.9 vs. 24.1; *p* = 0.002) compared to controls. We found that parents with PTSD showed FesEx (81.8 vs. 55; *p* = 0.022) and FesOrg (95.5 vs. 58.3; *p* = 0.001) items’ scores significantly higher compared to controls (Figure 1).

**TABLE 2:** Morbidity condition within hospital stay.

	Cases	Controls	*P* value
		
	*n* = 22	*n* = 60	
Invasive mechanical ventilation	11 (50.0)	26 (43.3)	*0.386*
Central vascular access	19 (86.4)	42 (70.0)	*0.109*
Parenteral nutrition	19 (86.4)	49 (81.7)	*0.447*
Nasal feeding tube	3 (13.6)	11 (18.3)	*0.447*
Phototherapy	11 (50.0)	34 (56.7)	*0.386*
Surgery	0 (0)	0 (0)	–
Patent ductus arteriosus	8 (36.4)	23 (38.3)	*0.541*
Intraventricular hemorrhage	2 (9.1)	13 (21.7)	*0.163*
Severe anemia	1 (4.5)	11 (18.3)	*0.108*
NEC	0 (0.0)	7 (11.7)	*0.102*

NEC, necrotizing enterocolitis; Data were expressed as *N* (%). Italic values represent the significance of *p*-value.

The binary logistic regression analysis was performed to evaluate the influence of confounding variables, that were significantly different at the univariate analysis (gestational age, parents’ age, Italian native, history of anxiety, history of depression, FesEx, FesOrg, Italian nationality, PSS:NICU sight and sounds, and KC), on the diagnosis of PTSD at 30 days postpartum, which revealed that the PTSD diagnosis was negatively significantly related (*p* < 0.01) to GA and KC, whereas it was positively significantly related (*p* < 0.01) to PSS:NICU sight and sounds (Table 3).

**TABLE 3:** Binary logistic regression analysis to evaluate the influence of covariates on the diagnosis of PTSD.

	β	Wald	S.E.	*P* value	OR (95% CI)
Gestational age	−2.678	4.227	1.303	0.040*	14.563 (1.133–187.14)
Parents’ age^a^	1.980	3.351	1.082	*0.067*	7.246 (0.869–60.391)
Italian native	1.517	1.364	1.299	*0.243*	4.560 (0.357–58.198)
History of anxiety	0.455	0.163	1.128	*0.687*	1.576 (0.173–14.379)
History of depression	2.151	1.786	1.610	*0.181*	8.596 (0.366–201.67)
FesEx^b^	0.593	0.356	0.995	*0.551*	1.810 (0.258–12.711)
FesOrg^c^	−0.144	0.020	1.018	*0.888*	0.866 (0.118–6.367)
PSS:NICU sight and sounds	2.433	5.408	1.046	0.020*	11.390 (1.466–88.512)
Kangaroo-Care	−2.619	5.910	1.077	0.015*	0.073 (0.009–0.602)

^a^Parents’ age > 35 years. ^b^FesEX, family environment scale expressiveness. ^c^FesOrg, Family Environment Scale Organization. *p < 0.05. *p < 0.01. Italic values represent the significance of *p*-value.

The multivariate analysis, including all the variables that were statistically significant in the univariate analysis, showed that the GA and intrauterine growth restriction (IUGR) were independently correlated with the occurrence of acute stress events (data not shown).

## Discussion

Parental PTSD following newborn NICU admission occurs in about 1/3 of parents. Thus, understanding the risk factors and preventive strategies for PTSD is a primary concern for optimal clinical care.

We found that PTSD was influenced, in newborns, admitted to NICU, by GA. The sight and sounds of the NICU significantly increase the risk of PTSD in parents of children observed in our unit. Whereas, the early application of KC within the first days of life, leading to parents’ participation in newborn’s care, could be considered a protective factor, that may contribute to decreasing the occurrence of PTSD.

Available evidence shows that parents who had NICU experience may have PTSD after their newborns’ premature birth (Ionio et al., 2016). However, recent studies reported that the severity of the child’s clinical condition at birth influences the occurrence of PTSD symptoms in parents (Pierrehumbert, 2003; Kersting et al., 2009). Previous studies did not demonstrate the direct relationship between GA and PTSD. We did not find a relationship between the occurrence of morbidity related to premature birth and PTSD. Thus, it seems that the clinical evolution of newborns, admitted to NICU, is not as important as the beginning condition of the neonate, which represents the main stress trigger probably because parents may not have been able to prepare themselves for separation from the baby, independently of the severity of his clinical condition. Moreover, we observed that the main risk factor that had an impact on the occurrence of acute stress was GA and IUGR, underlining how parental stress was strongly associated with the prenatal condition of the baby. Given that, the occurrence of the disorder in the parents relies mainly on the concern of premature birth, considering that the less GA, itself, poses parents into a worry mood that, if not well handled, may increase the risk of PTSD.

The stressful nature of the NICU environment for parents of ill newborns has been previously reported (Miles and Holditch-Davis, 1997). We have demonstrated that among all the environmental stress factors included in the PSS:NICU scale, sights and sounds factor (i.e., machines, equipment, lights, noises, infants, and staff) is an independent physical environment inducing PTSD. According to international recommendations, the critical environment stress factor, characterized by unpredictable and loud sounds and unpleasant sights, may be prevented, through the application of specific strategies (White et al., 2013). Hospitals first must thoroughly assess the noise levels in their NICUs and perform either the implementation of a quiet hour, as suggested by Strauch et al. (1993) including training of nurse and medical staff behavior in terms of either modulating the level of the voice bed-side, or the covering of the equipment or devices when not necessary for procedures and adjusting the interpersonal relationships between family members and staff in a family-centered way (Bremmer et al., 2003).

The beneficial effects of KC on preterm health are well acknowledged (Nyqvist et al., 2010). A growing body of literature has examined the impact of skin-to-skin care on parents’ mental health. Research in the field showed that parents who chose to provide KC had less anxiety and depressive symptoms, were more sensitive and less intrusive, showed more attachment behaviors, and had shorter latencies to joint attention with their infants than parents not providing KC. Inasmuch, infants receiving KC were less negative and more alert (Feldman et al., 2003, 2014; Tallandini and Scalembra, 2006; de Alencar et al., 2007). However, in all those studies, parents’ experiences have not been evaluated using standard tools. The direct relation between KC and PTSD of parents has not been investigated, and neither the PTSD diagnosis has been made adopting PTSD-specific scale measures. If studies demonstrated the findings that KC can be an effective intervention to reduce anxiety and improving symptoms of postpartum depression and general aspects of mood (Scime et al., 2019), then there are other studies which have not found an impact of KC on maternal stress during the NICU stay (Roberts et al., 2000). Further studies found that KC increased the rate of decline in maternal worry but did not affect maternal distress (Holditch-Davis et al., 2013). Even in those studies, parents’ stress has not been evaluated using PTSD scale diagnosis. We diagnosed PTSD in all parents included in the study through standard criteria. We studied the specific relationship between KC and PTSD, using CAPS scale measures. The CAPS, a 30-item structured interview, is generally recognized as the gold standard in the PTSD assessment (Weathers et al., 2018). Thus, we believe that the implementation of KC, in the very first days of life, is a protective tool against the occurrence of PTSD in subjects at high risk. In agreement with some studies that demonstrated a reduction of cortisol levels in parents involved in KC, we can speculate that the protective role of KC in reducing PTSD might relies on the fluctuation of cortisol level in parents during KC. These results may represent a pathophysiological explanation of the reduction of PTSD associated with KC exposure. In particular, Vittner et al. (2018) showed reduced parent anxiety scores and parent cortisol levels during skin-to-skin contact; however, the specific relationship between parents’ stress, evaluated by the PTSD scale, and KC was not investigated. Similarly, Cong et al. (2015) found that maternal cortisol continuously decreased after KC, however, also in this study, the association between KC and stress was not investigated; moreover, the parents’ stress has not been evaluated through a standardized scale, but only in terms of cortisol reduction.

Despite being interesting, our study should be interpreted according to study limitations.

First, the study is not blinded and physicians administering scale measures to newborns’ parents were not blinded, hence this might have led to a selection bias. Second, confounding variables still unknown, or not considered in our statistical model, may have influenced the results. We adjusted results for confounding variables that could have influenced the outcome of the study. An important drawback of the study was the convenience sample (homogeneity): all participants were recruited in the same university hospital and had to speak Italian, thus limiting the generalizability of the findings. Our results may be different in other centers. Further studies are necessary to better address this issue.

## Conclusion

The occurrence of PTSD in parents of newborns admitted to NICU is a pathological condition that should be taken into account, and properly managed, in the very first days after a traumatic birth. Nevertheless, this disorder is seldom used as an outcome measure of NICU interventions and strategies. To limit parents’ PTSD, implementation of specific strategies should be adopted. First, clinical staff after gathering parents’ perceptions should provide accurate information about prematurity and its consequences with attentive communication, giving the parents relief and limiting unexpected reactions after birth. Moreover, to reduce environmental stress, it would be appropriate to create a family environment in which babies and parents can feel welcomed and protected. Besides, parents and their newborns may be considered as a family unit, thus the inclusion of the parents, as soon as possible, in neonates’ care should be a step to address. The KC has been demonstrated to be a protective factor in the occurrence of PTSD, therefore it should be adopted in the parents’ involvement approach. Finally, given that NICU hospital stay requires quite some time for parents to spend in a foreign environment, clinical staff must improve their communication skills, gather parents’ perceptions, and understand their needs and worries. It might be valuable considering the experience of NICU admission as a whole family matter, not just a condition affecting the baby; thus, the promotion of a family-centered intervention, starting from gathering daily parents’ perspectives and ensuring a continuative parents’ support during the hospital stay may be useful. The very first days of neonatal life are decisive for applying prevention strategies and reducing parents’ stress. Further studies are advocated to eventually identify other risk factors involved in the PTSD-NICU diagnosis leading to better strategies implementation preventing that tough condition.

## Data availability statement

The raw data supporting the conclusions of this article will be made available by the authors, without undue reservation.

## Ethics statement

Ethical review and approval was not required for the study on human participants in accordance with the local legislation and institutional requirements. Written informed consent to participate in this study was provided by the participants’ legal guardian/next of kin.

## Author contributions

GT, MDC, and GL: design of the study, continuous supervision of the study, and periodic discussion of ongoing results. GT, MDC, GL, and BD: development of laboratory methods, statistical analysis of results, and quality control. MDC and GL: selection of patients, their follow-up, and treatment (together with GT). All authors contributed to the article and approved the submitted version.
