# Supplementary material for: Risk factors and preventive strategies for post-traumatic stress disorder in neonatal intensive care unit
Source: Front Psychol. 2022 Nov 9;13:1003566. doi: 10.3389/fpsyg.2022.1003566 (PMC9683535; doi:10.3389/fpsyg.2022.1003566)
Supplement: Supplementary file 1 [file Data_Sheet_1.DOCX]

**Supplementary materials**

1. *Kangaroo-care intervention protocol*

KC involved holding the infant in skin-to-skin contact in an upright position between the mother’s breasts. The infant is dressed only in a diaper and hat, and the side of the infant not in contact with the mother is covered. During KC, the infant usually sleeps but may suck or feed at the breast (9,13). Mothers or fathers were instructed to do KC for as long as they wanted but for at least 15 min., and to stop it if the infant’s temperature fell or if apnea or bradycardia occurred.

Each dyed mother/father and newborn was monitored by a trained nurse, caring for the newborn.

1. *The Clinician-Administered PTSD Scale (CAPS)*

CAPS is an extensively validated and widely used structured diagnostic interview for PTSD^33^. It has been recently revised to correspond with PTSD criteria of the DSM 5^th^ Edition. It consists in a 30-item structured interview assessing the 20 DSM 5^th^ Edition PTSD symptoms, the onset and duration of symptoms, the impact of symptoms on social and occupational functioning, and the overall PTSD severity (14). CAPS-5 items are rated with a single severity score, in contrast to previous versions in which separate frequency and intensity scores were required (14). The parent is asked to rate each item on a 5-point scale from absent (0) or mild/subthreshold (1) to extreme/incapacitating (4). Previous studies demonstrated high internal consistency and test-retest reliability for CAPS-5 total severity score (10,15). We used CAPS-5 past month version to make current diagnosis of PTSD among parents 30-days after the infant’s admission to the NICU, thus, it was used as an outcome measure after intervention (10).

1. *Parental Stressor Scale (PSS:NICU)*

The PSS:NICU is a 26-item self-report scale that measures parental anxiety and stress (6) There are three factors structure: sights and sounds (SS), Infant behavior and Appearance (IBA), Parental Role Alteration (PRA). The parent is asked to rate each item on a 5-point Likert type scale from not experienced (0) or not stressful (1) to extremely stressful (5). Three metrics can be calculated for the PSS:NICU: 1) Stress Occurrence Level, which calculates the stress score using only those items that parents indicate as a source of stress; 2) Overall Stress Level, which calculates the stress score using all the items, giving a rating of 1 (not at all stressful) for those items not experienced by parents; 3) Frequency of items experienced by parents. The mean subscale and total scores can be considered for clinical, and research aims. The tool has demonstrated excellent internal consistency and test-retest reliability in North American and UK NICUs. We used PSS:NICU to assess parental anxiety and stress related to NICU environment in parents of enrolled infants at baseline (5,6).

PSS:NICU scores were considered pathological when SS, IBA and PRA > 3 points and PSS global > 9 points.

1. *The Family Environment Scale (FES)*

FES scale measures the social environment of all types of families. It is composed of ten subscales or dimensions (16)Which are divided into three sets: the Relationship Dimensions, the Personal Growth Dimensions, and the system Maintenance Dimensions. Relationship Dimensions asses how involved people are in their family and how openly they express both positive and negative feeling. It includes 3 subscales Cohesion (FesC), Expressiveness (FesEX), Conflict (FesCon). The Personal Growth Dimensions is a set focused on the family’s goals by tapping the major ways in which a family encourages or inhibits a personal growth. It includes five subscales: Independence (Ind), Achievement Orientation (AO), Intellectual-Cultural Orientation (ICO), Active-Recreational Orientation (ARO), Moral-Religious Emphasis (MRE). The System Maintenance Dimensions asses the family’s emphasis on clear organization, structure, rules, and procedures in running family life. There are 2 subscales in this set: Organization (Org) and Control (Ctl) (16,17).

FES scores were considered pathological when FesC ≥8; FesEx ≥7; FesCon ≥6; FesInd ≥6; FesAO ≥7; FesICO ≥7; FesARO ≥7; FesMRE ≥6; FesOrg ≥7; FesCtl ≥6.

1. *Spielberger State—Trait Anxiety Inventory (STAI-S; STAI-T)*

The STAI consists of two parts measuring the State (response to present situation) and Trait (pre- disposition to be anxious). anxieties separately. Each section comprised of 20 questions and each question has been scored 1 to 4. For the state items, respondents are asked to indicate “how you feel right now, that is, at this moment”. Responses indicate intensity of feeling on a 1 to 4 scale, from “not at all” through “somewhat”, “moderately so” to “very much so”. For the trait items the question concerns “how you generally feel” and the response scale indicates frequency as “almost never”, “sometimes”, “often” and “almost always “. The total score for each individual will be between 20 and 80 (18). STAI scores were considered pathological when > 20 points.

1. *Beck Depression Inventory Second Edition (BDI-II)*

The BDI-II is a 21-item questionnaire administered at baseline and follow- up, assesses depressive symptoms with a reliability of 0.92 (12). It is the most thoroughly researched and used self-report measure of depression in both clinical and non-clinical populations (19). BDI-II score was considered pathological when > 44 points.
